# When old metagenomic data meet newly sequenced genomes, a case study

**DOI:** 10.1371/journal.pone.0198773

**Published:** 2018-06-14

**Authors:** Xin Li, Saleh A. Naser, Annette Khaled, Haiyan Hu, Xiaoman Li

**Affiliations:** 1 Department of Computer Science, University of Central Florida, Orlando, Florida, United States of America; 2 Burnett School of Biomedical Science, College of Medicine, University of Central Florida, Orlando, Florida, United States of America; Oklahoma State University, UNITED STATES

## Abstract

Dozens of computational methods are developed to identify species present in a metagenomic dataset. Many of these computational methods depend on available sequenced microbial species, which are still far from being representative. To see how newly sequenced genomes affect the analysis results, we re-analyzed a shotgun metagenomic dataset composed of twelve colitis free metagenomic samples and ten colitis-related metagenomic samples. Unexpectedly, we identified at least two new phyla that may relate to colitis development in patients, together with the phylum identified previously. Compared with the previously identified phylum that differed between the two types of samples, the differences associated with the two new phyla are statistically more significant. Moreover, the abundance of the two new phyla correlates more with the severity of colitis. Surprisingly, even by repeating the analyses implemented in the previous study, we found that at least one main conclusion in the previous study is not supported. Our study indicates the importance of re-analysis of the generated metagenomic datasets and the necessity of considering multiple updated tools in metagenomic studies. It also sheds light on the limitations of the popular tools used currently and the importance to infer the presence of taxa without relying upon available sequenced genomes.

## Introduction

A plethora of metagenomic datasets have been generated in the past fifteen years [1–4]. Early datasets are often based on 16S rRNA profiling and Sanger sequencing [5–7]. Later datasets are usually sequenced by next generation sequencing technologies [8, 9]. The generated datasets vary from the early ones such as those in seawater [2], acid mine drainage [10], and deep sea [11, 12] to current ones such as those in gut [8, 13], skin [14], soil [15], etc. These metagenomic datasets have enabled an unprecedented exploration of microbes, which has significantly advanced our understanding of microbes in the living world [3, 4, 8].

Correspondingly, dozens of computational methods are developed for the analyses of metagenomic datasets. These include methods for filtering erroneous and duplicated reads, methods for gene prediction directly from metagenomic reads, similarity-based and abundance-based methods for read binning, methods for contig binning and genome assembly, etc. [16–30]. These methods altogether have significantly advanced our understanding of the genetic contents in various metagenomic datasets [31–33].

The majority of available computational methods that perform on metagenomic datasets somewhat rely upon available sequenced genomes. For instance, most methods predict species present in metagenomic datasets depend on the annotation of the available sequenced genomes, such as Megan and MetaPhlAn [16, 17]. Megan is an early method that infers species presence based on the comparison of shotgun metagenomic reads with annotated sequences [16]. MetaPhlAn is a popular method for inferring species present in a metagenomic dataset with marker genes, which infers marker genes from sequenced genomes [17]. It is understandable that most methods are based on annotated genomes, as more information is taken into account in the analyses and thus more reliable conclusions may be made. Moreover, although metagenomic reads can be studied and analyzed without sequenced genomes, such as read binning and gene prediction, the automatic inference of the origin of a sequence and the presence of a species without any prior information is still infeasible.

Dubin et al. generated a metagenomic dataset to study colitis development in metastatic melanoma patients followed by CTLA4-blockage [34]. In their study, shotgun metagenomic reads are sequenced from faecal samples of each of twelve colitis-free (CF) patients and each of ten progressed to colitis (PtC) patients, together with 16S rRNA reads sequenced from faecal samples of each of 34 patients. These 22 patients from whom the shotgun metagenomic reads came are included in the 34 patients used for 16S rRNA sequencing. This study pointed out that taxonomical analysis results based on 16S rRNA reads from the 34 samples were similar to those based on shotgun metagenomic reads from the 22 samples by the popular method MetaPhlAn [34]. In brief, the phylum *Bacteroidetes* and its three families, *Bacteroidaceae*, *Rikenellaceae*, *and Barnesiellaceae*, were identified to be significantly enriched in CF samples compared with PtC samples (Mann-Whitney test p-value 0.013 for *Bacteroidetes*, p-value 0.007, 0.023 and 0.013 for the three families, respectively). For simplicity’s sake, we used “between samples” to refer to “between CF samples and PtC samples” in the following. Moreover, the abundance of reads from *Bacteroidetes* and its three families negatively correlates with the severity of colitis, with the Spearman’s rank correlation coefficient around -0.38, -0.43, -0.42 and -0.43, respectively.

Since this original study was published two years ago [34], genomes of more microbes have been sequenced. Moreover, MetaPhlAn, the tool used in this study, is based on marker genes, which cannot fully utilize the information buried in metagenomic reads [17]. We thus re-analyzed all shotgun metagenomic reads generated from the 22 patient samples in this metagenomic dataset by mapping reads to all sequenced microbial genomes instead of considering only reads from marker genes (Methods). We considered shotgun metagenomic reads only, as they are more unbiased for taxonomical analysis than 16S rRNA reads [35, 36]. Unexpectedly, we found that reads from *Bacteroidetes* are only marginally more in CF samples than in PtC samples. Moreover, significantly more reads from at least two new phyla, *Thaumarchaeota* and *Actinobacteria*, are in PtC samples than in CF samples. The abundance of reads from these two new phyla correlates with the severity of colitis much better than that from *Bacteroidetes*. By further studying low level taxa based on different strategies, we found that the read abundances of at least 2 classes, 9 orders, 22 families, 70 genera, and 162 species are significantly different between the two types of samples, and correlate with the severity of colitis in patients better than that of *Bacteroidetes*. Surprisingly, by repeating the analysis performed in the original study on this dataset with both old and current versions of MetaPhlAn [34], we found that the previously identified phylum *Bacteroidetes* is not significantly different between samples while one of the newly identified phyla, *Actinobacteria*, is identified as the only significant phylum between the samples. Our study demonstrated the necessity to reanalyze the generated metagenomic data, the limitation of the marker gene based methods, and the importance of being cautious about the inference from available sequenced genomes in metagenomic studies.

## Results

### At least two new phyla may relate to colitis development in patients

**Fig 1:**
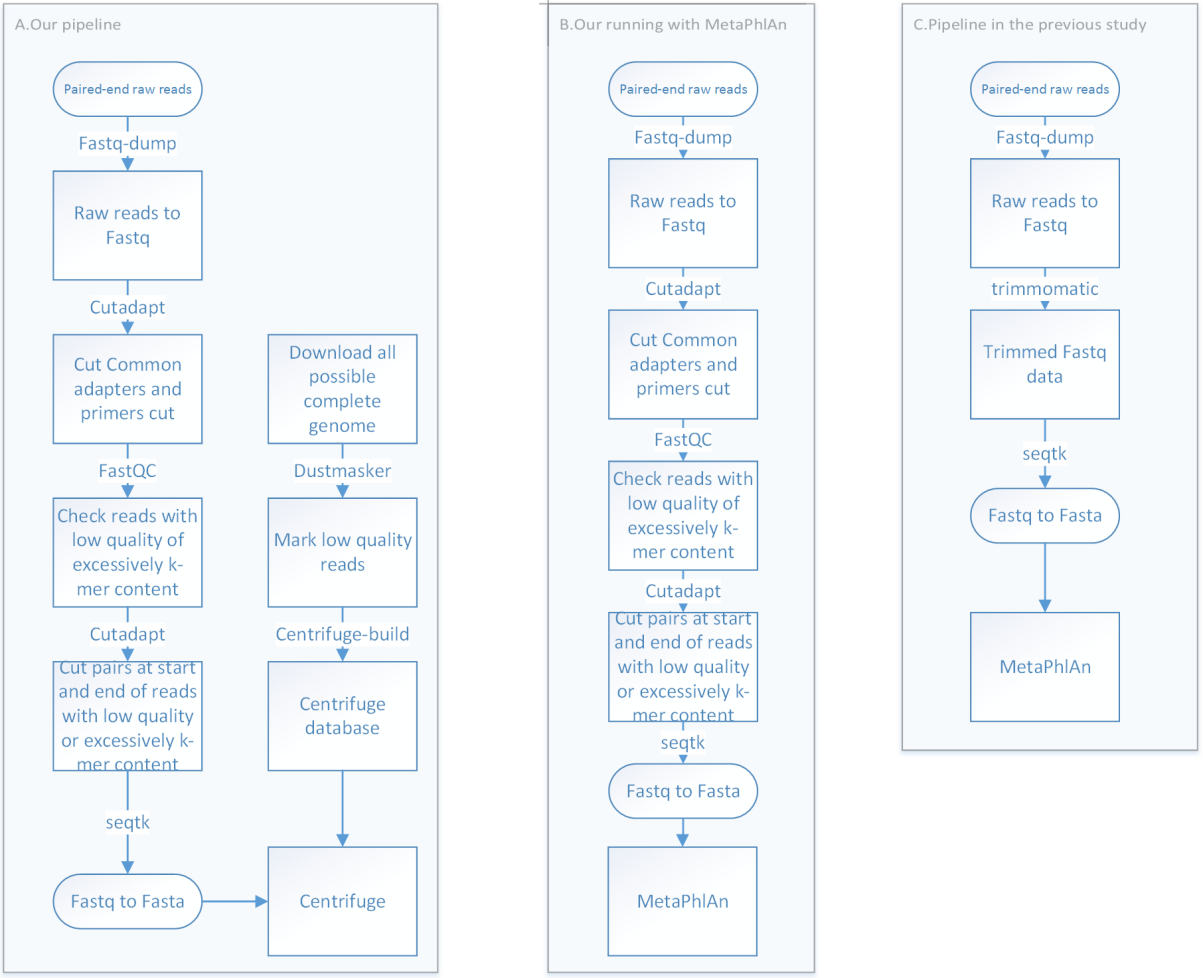
Pipelines to analyze shotgun metagenomic reads. The left panel shows our pipeline. Centrifuge outputs the reads mapped to the sequenced genomes, from which the read abundances of taxa, Mann-Whitney p-values, and correlations with colitis severity are calculated. The middle panel shows the analyses with MetaPhlAn by a different read trimming procedure from that in the original study. The right panel shows the pipeline using MetaPhlAn in the original study. As it is not clear which MetaPhlAn version was used in the original study, two versions of MetaPhlAn have been used for comparisons in this study.

**Fig 2:**
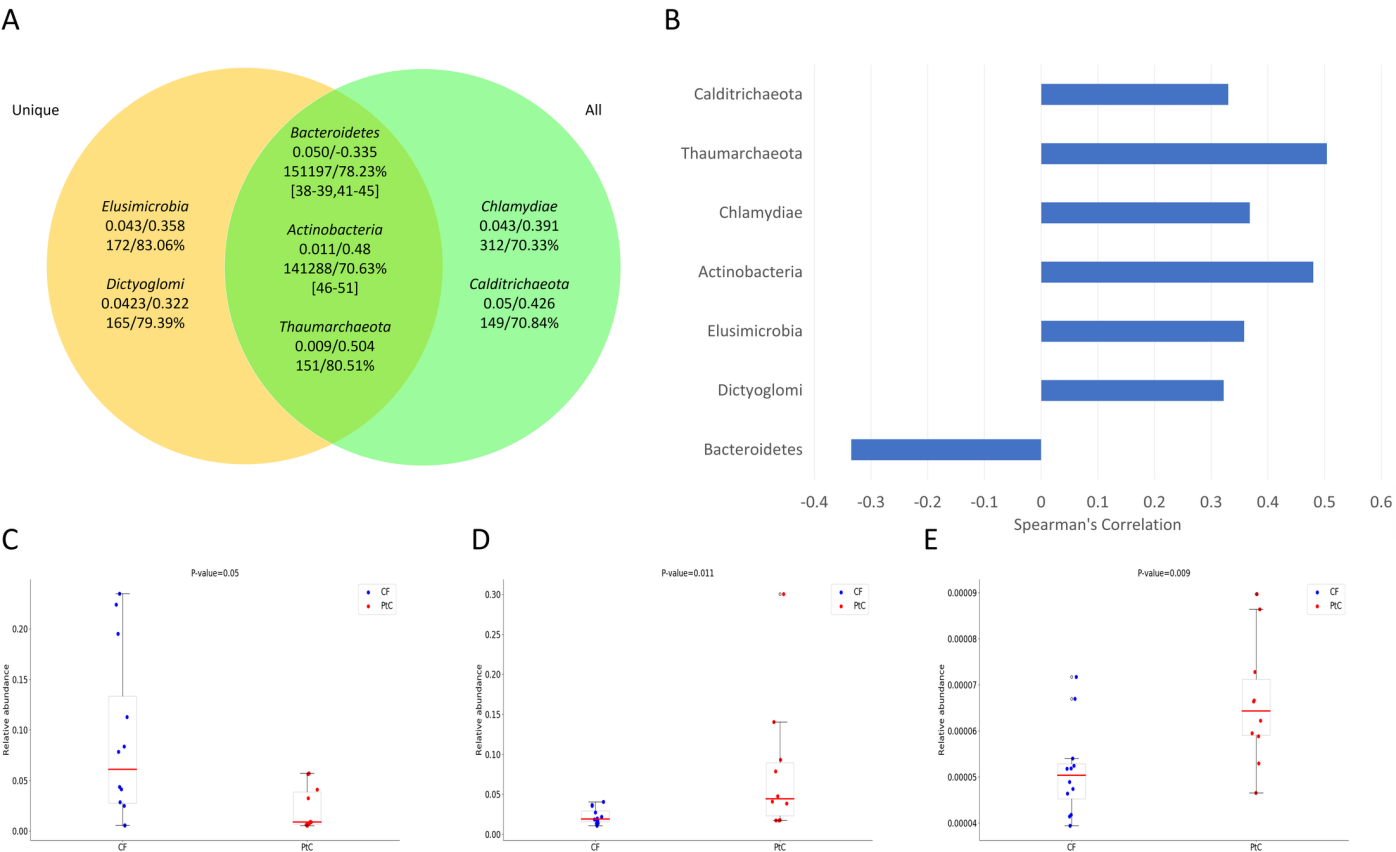
Significant phyla from unique reads only and all mapped reads, respectively. A. Seven significant phyla identified. The two numbers below a phylum name are the Mann-Whitney p-values, the Spearman’s correlations of the read abundances with the colitis severity. The next row provides the average number of reads mapped to each of the 22 samples and the percentage of unique reads among all mapped reads for the phylum. The third row below a phylum name gives the references that may support the colitis-relatedness of this phylum and its lower taxa. B. The Spearman’s correlation for seven phyla. C-E: the scatter plot of the relative read abundances in *Bacteroidetes*, *Actinobacteria*, and *Thaumarchaeota*, respectively.

We mapped shotgun metagenomic reads from each of the 22 faecal samples to about 15,000 sequenced microbial genomes and compared the relative abundance of reads from every phylum in CF samples with that in PtC samples (Fig 1 and Methods). We discovered that the abundance of reads from seven phyla are significantly different between CF samples and PtC samples (Mann-Whitney p-value < = 0.05), including *Bacteroidetes* identified previously [34]. Five phyla were identified when only uniquely mapped reads were considered. A different set of five phyla were identified when both unique and multi-mapped reads were considered (Fig 2A). Multi-mapped reads are reads that can be mapped to multiple sequenced microbial genomes. For convenience, we call multi-mapped reads and uniquely mapped reads multi-reads and unique reads, respectively.

With unique reads, we identified five phyla that are significantly different between samples. They are *Thaumarchaeota* (p-value = 0.009), *Actinobacteria* (p-value = 0.011), *Dictyoglomi* (p-value = 0.043), *Elusimicrobia* (p-value = 0.043), and *Bacteroidetes* (p-value = 0.050) (Fig 2A). Although *Bacteroidetes* discovered in the original study is identified, it has the largest Mann-Whitney test p-value, suggesting that the four new phyla are even more significant and may be more related to colitis development. In fact, *Bacteroidetes* has a negative correlation of -0.335 with the severity of colitis, while *Thaumarchaeota*, *Actinobacteria*, *Elusimicrobia*, and *Dictyoglomi* have a similar or higher positive correlation of 0.504, 0.480, 0.358, and 0.322, respectively (Fig 2B).

We also studied significant phyla with all mapped reads (i.e., unique reads and multi-reads) (Methods). We identified the following five phyla that are significantly different between samples: *Thaumarchaeota* (p-value = 0.014), *Actinobacteria* (p-value = 0.021), *Chlamydiae* (p-value = 0.043), *Calditrichaeota* (p-value = 0.050), and *Bacteroidetes* (p-value = 0.050) (Fig 2A). *Bacteroidetes* again is not as significant as three of the other four new phyla. The four new phyla also have a higher positive correlation with the colitis severity than *Bacteroidetes*. The correlations of the read abundances with the severity of colitis for *Thaumarchaeota*, *Actinobacteria*, *Chlamydiae*, *Calditrichaeota*, and *Bacteroidetes* are 0.426, 0.484, 0.391, 0.426, and -0.335, respectively. Three phyla (*Thaumarchaeota*, *Actinobacteria*, and *Bacteroidetes*) are identified with unique reads only and with all mapped reads as well, suggesting that at least three phyla may relate to colitis development in patients (Fig 2). The p-value of *Thaumarchaeota* and *Actinobacteria* is changed with all mapped reads compared with only unique reads (Fig 2A), indicating the difference of the abundance of multi-reads relative to unique reads between the two types of samples for these two phyla.

Several aspects are different between *Bacteroidetes* and the six new phyla. First, there are many more reads mapped to Bacteroidetes than to other phyla. In each of the 22 samples, *Bacteroidetes* on average has 151,197 mapped reads, while the six new phyla except *Actinobacteria* on average have fewer than 320 mapped reads (Fig 2A). Since the original study applied a marker gene based method to identify significant phyla and it is unlikely that the small number of sequenced reads from the five new phyla come from marker genes, it is not surprising that it missed these five low abundance phyla. In terms of *Actinobacteria*, which has 141,288 mapped reads on average in each sample, the latest version and the old version of MetaPhlAn indeed identify this phylum as significant (see the fourth results section). The original study did not report this phylum, maybe because the 16S rRNA read analysis did not show the significance of this phylum. Second, PtC samples have more reads from the six new phyla than CF samples, while it is opposite for *Bacteroidetes* (Fig 2B). Third, except *Dictyoglomi*, the abundance of reads from new phyla have more significant correlations with the severity of colitis than that from *Bacteroidetes* (Fig 2B).

In summary, at least three phyla (*Bacteroidetes*, *Actinobacteria*, *Thaumarchaeota*) are highly likely related to colitis development in patients (Fig 2B–2E). The read abundances of *Thaumarchaeota* and *Actinobacteria* are more different between samples compared with *Bacteroidetes* based on only unique reads and all mapped reads (Fig 2C–2E). Moreover, their abundances correlate with the severity of colitis better than that of *Bacteroidetes* (Fig 2B). In addition, *Elusimicrobia* and *Chlamydiae* may be related to colitis development in patients as well. This is because their properties of read abundances and correlations are similar as the above three phyla, although they are not identified by both all mapped reads and unique reads only. It is worth pointing out that there is at least one significant lower level taxon identified by unique reads from each of these five phyla, as shown in the next section.

### Hundreds of lower taxa may relate to colitis development in patients

We further compared read abundances from lower taxa between samples (Methods). If we consider only unique reads, there are 3 classes, 14 orders, 34 families, 101 genera and 244 species with read abundances different between samples (p-value< = 0.05) (S1 Table). If we consider all mapped reads, 6 classes, 15 orders, 43 families, 116 genera and 334 species have different read abundances between samples (p-value< = 0.05) (S2 Table). In total, there are 7 classes, 20 orders, 52 families, 143 genera and 406 species with read abundances different between samples (Tables 1 and S1 and S2). Note that due to the large number of un-sequenced genomes, when the read abundances of a taxon is significantly different between samples, the read abundances of neither its ancestral taxa nor its offspring taxa may be significantly different between samples.

**Table 1:** The number of taxa identified based on different criteria.

The taxon level	#taxa from unique reads	#taxa from all mapped reads	#taxa from unique or all mapped reads	#correlated taxa from unique reads	#correlated taxa from all mapped reads	#correlated taxa from unique or all mapped reads	#correlated taxa from both unique reads and all mapped reads
phylum	5	5	7	4	5	6	3
class	3	6	7	2	6	6	2
order	14	15	20	12	14	17	9
family	34	43	52	28	40	46	22
genus	101	116	143	95	109	134	70
species	244	334	406	221	309	368	162

**Fig 3:**
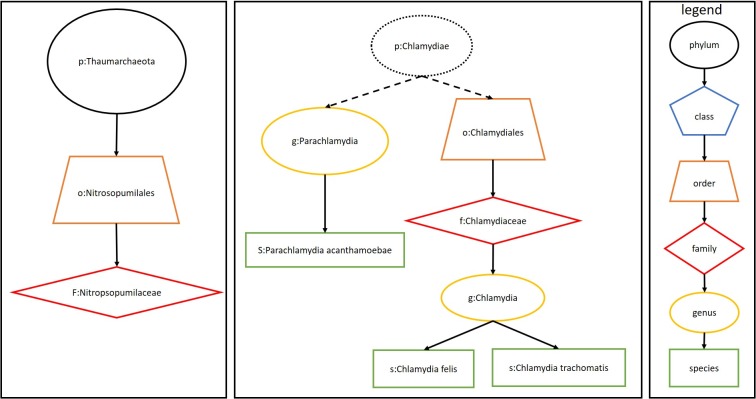
Lower taxa identified from the phyla *Thaumarchaeota* and *Chlamydiae*. Only taxa from the last column of Table 1 are shown. Note that no class from these two phyla are identified in the last column of Table 1. The phylum *Chlamydiae* is presented in a dotted box, as it is not identified in the last column of Table 1.

The aforementioned five phyla that may relate to colitis development (*Thaumarchaeota*, *Actinobacteria*, *Elusimicrobia*, *Bacteroidetes*, and *Chlamydiae*) all have lower taxa that are significantly different between samples based on unique reads. *Bacteroidetes* has four families, nine genera, and nineteen species with read abundances significantly different between samples. Two of the four families, *Rikenellaceae* and *Barnesiellaceae*, which were reported in the original study, are significantly different between samples. Although the abundance of reads from *Bacteroidetes* itself negatively correlates with the colitis severity, the read abundances from some of its significant lower level taxa positively correlates with the colitis severity. For instance, the species *Bacteroides caccae* has a p-value of 0.006 and a negative correlation of -0.468, while the species *Blattabacterium sp* has a p-value of 0.043 and a positive correlation of 0.438. *Actinobacteria* has eleven species, two genera, one family, one order and one class with read abundances significantly different between samples. All these lower taxa are all under the class *Actinobacteria*, which is the class for high G+C Gram-positive bacteria but is not significant itself, implying that certain Gram-positive bacterial species may play an important role in PtC patients. The two phyla, *Thaumarchaeota* and *Elusimicrobia*, each has one significant species and at most one significant lower taxon at every lower taxonomical level (Fig 3). For instance, *Elusimicrobia* has only one class, one order, one family, one genus, and one species with read abundances different between samples. The remaining phylum, *Chlamydiae*, has one order, one family, two genera and three species with read abundances significantly different between samples (Fig 3).

In terms of correlation with the severity of colitis in patients, the read abundances of these significant lower taxa of the above five phyla based on unique reads has higher correlation than that of *Bacteroidetes* (S1 Table). *Bacteroidetes* has three families, eight genera and sixteen species with higher correlations than *Bacteroidetes* itself. Among the three families, *Rikenellaceae* (p-value 0.021, correlation -0.463) and *Barnesiellaceae* (p-value 0.025, correlation -0.381) are also identified in the original study, while the family *Dysgonamonadaceae* (p-value 0.05, correlation-0.342) is missed by the original study. Among all lower taxa, four genera and eight species have positive correlations with the colitis severity, while the remaining eight species, four genera, and three families have negative correlations. The average of these negative and positive correlations is -0.390 and 0.459, respectively. *Actinobacteria* has ten species, two genera, one family, one order and one class with higher correlations with the severity of colitis, and the average of these correlations is 0.447. *Thaumarchaeota* has a significant family and a significant order with an average correlation of 0.478 when we consider only unique reads. *Elusimicrobia* has one significant lower taxon with the correlation of 0.402 at each taxonomical level. *Chlamydiae* has one order, one family, two genera, and three species with an average of correlation of 0.414.

When we consider all mapped reads, the five phyla discussed above also have lower taxa that are significantly different between samples, and their abundance has higher correlations with the severity of colitis than that of *Bacteroidetes* (S2 Table). *Bacteroidetes* has three families, nine genera and seventeen families that are significant and have higher correlations. The average negative correlations of lower taxa is -0.381 and the average of positive correlations is 0.433. *Actinobacteria* has one class, one genus and ten species that are significant and have an average correlation of 0.456. The same as the lower taxa from unique reads, ten species are from the same class of *Actinobacteria*, which is the class for gamma-positive bacteria and is not significant itself. Another class, *Coriobacteriia*, is opposite to the *Actinobacteria* class, in that the class itself is significantly different between the two groups and its abundance correlates better with the severity of colitis while this class has no significant lower taxon. *Elusimicrobia* has exactly one lower taxon at each level with the abundance significantly different between the two groups and correlating better with the severity of colitis. The average correlation of significant lower taxa is 0.457. *Thaumarchaeota* has one class, two orders, two families, one genera and one species that are significant and their abundance correlate better with the severity of colitis. The average correlation is 0.396. The class under the *Thaumarchaeota* phylum, *Nitrososphaeria*, has exact one taxon at each of its lower levels. *Chlamydiae* has one class, one order, two families, two genera and four species that are significant and have higher correlations with the severity of colitis, and the average correlation is 0.410. Among them, *Chlamydia felis* is the species identified with the highest correlation of 0.587 in this study.

It is also worth pointing out that although we focus on lower taxa under the five phyla, there are many significant lower taxa not from these five phyla (S1 and S2 Tables). For instance, there are at least 8 orders, 21 families, 82 genera and 192 species that do not belong to the five phyla with their abundance significantly different between the two types of samples and correlate better with the severity of colitis than *Bacteroidetes*.

A large proportion of the significant lower taxa from unique reads and from all mapped reads are the same (Tables 1 and S1 and S2). For all significant lower taxa, we have identified 2 (42.86%) classes, 9 (28.57%) orders, 25 (45.00%) families, 74 (51.75%) genera and 172 (42.36%) species from both unique reads only and from all mapped reads. All five phyla have at least one shared lower taxon from both unique reads only and from all mapped reads, and the lower taxa have higher correlations than their parent taxa. Among all these significant lower taxa, the abundance of 2 (100%) classes, 9 (100%) orders, 22 (88.00%) families, 70 (94.59%) genera, 162 (94.19%) species correlate with the severity of colitis better than that of *Bacteroidetes* (Table 1, last column). We believe that these taxa are highly likely colitis-related taxa.

### Many identified taxa may relate to colitis development based on literature

Microbes are known to play a vital role in the development of colitis [37]. We thus studied whether the above taxa are colitis related based on literature, since their abundance is significantly different between samples and correlates better with the severity of colitis than *Bacteroidetes*. Because the number of these taxa is large, we focused on the three most confident phyla (*Bacteroidetes*, *Actinobacteria*, *Thaumarchaeota*) and their lower taxa. We found that at least 2 (66.7%) of the three phyla and 11 (42.31%) of the identified species in the three phyla are likely colitis related.

We found that microbes from at least two of the three phyla are showed to be related to colitis [38–40]. Bacteria from *Bacteroidetes* belong to Gram-negative bacteria [41], which are known risk factors for inflammatory bowel diseases such as colitis [42–45]. The phylum *Actinobacteria* belongs to Gram-positive bacteria. Gram-positive commensal bacteria induce colitis by recruiting colitogenic monocytes and macrophages [46]. *Actinobacteria* was found increasingly in abundance in colitis groups compared with control non-colitis groups in different experiments as well [47–50].

There are also four and seven lower taxa that may relate to colitis in the phyla *Bacteroidetes* and *Actinobacteria*, respectively. In term of *Bacteroidetes*, Ye et al. analyzed faecal samples collected from patients with colitis and found that the abundance of *Barnesiella viscericola* correlates with the disease activity in IL-10^-/-^ mice [39]. *Barnesiella viscericola* are found by unique reads with the Mann-Whitney p-value of 0.025 and its abundance has a correlation coefficient of -0.381 with the severity of colitis. Another example is the *Bacteroides*, whose abundance is significantly different between samples and correlates with the colitis severity better than *Bacteroidetes*. *Bacteroides* are found to be accumulated in inflamed ileum at high concentrations [38]. For all sixteen species in *Bacteroidetes* with their abundance significantly different between PtC samples and CF samples as well as correlating better with the severity of colitis than *Bacteroidetes*, three species are from the genus *Bacteroides*. They are *Bacteroides caccae* (p-value 0.006, correlation -0.468), *Bacteroides salanitronis* (p-value 0.025, correlation -0.383) and *Bacteroides cellulosilyticus* (p-value 0.036, correlation -0.359). As to the phylum *Actinobacteria*, the analyses of the microbiota in mucosa of patients with ulcerative colitis (UC) show that there are more *Actinobacteria* and *Proteobacteria* in patients compared with controls [47]. Especially, microbiota of patients with UC have high level of abundance of the genus *Rhodococcus* and a low abundance of both *Bacteroides* and *Prevotella* genera compared with the controls. We found that the abundance of the species *Rhodococcus erythropolis* under the *Rhodococcus* genus is significantly different between samples (p-value 0.036, correlation 0.421). Another study also indicates that species in *Rhodococcus* causes infection in patients [51]. Rooks et al. found that gut microbiomes of colitis patients were most significantly enriched in *Actinobacteria*, including *Corynebacterium*, compared with the controls [50]. Four species we identified in this study are from *Corynebacterium* and have an average correlation of 0.481 with the severity of colitis.

Besides the significant taxa related to colitis from the three most confident phyla, there are other taxa supported by literature (S1 and S2 Tables). These are in total 101 taxa under the phylum *Firmicutes* [40, 45, 47, 48, 50–53], 3 under the phylum *Proteobacteria* and 1 under the phylum *Fusobacteria* [40]. Among them, the majority of taxa are actually lower level of the *Bacillales* order, which includes 80 of the lower taxa we identified (the order *Bacillales* itself, 4 families, 16 genera and 59 species). Rooks et al. demonstrate that *Bacillales* plays an important role in colitis in gut [50]. They also found the genus *Staphylococcus* are more enriched in colitis patients [50]. In our study, we found that the abundance of ten taxa (*Staphylococcus* itself and nine species) from *Staphylococcus* are significantly different between samples and correlates well with the colitis severity.

### Re-analyses with MetaPhlAn support that *Actinobacteria* is different between CF samples and PtC samples

The original study generated and analyzed the same shotgun metagenomic dataset with MetaPhlAn [34]. From the original study, they concluded that the read abundances of *Bacteroidetes* and its three families *Bacteroidaceae*, *Rikenellaceae* and *Barnesiellaceae* are significantly different between samples, and correlate well with the severity of colitis. Since we cannot find the list of all taxa this study identified, especially their analyses results from the shotgun metagenomic reads, which was only partially shown in their S2 Fig [34], we followed their procedure and applied the 1.7.7 version and the 2.1.0 verion of MetaPhlAn to the same shotgun metagenomic data (Fig 1). The only difference we made is that we further trimmed reads with Cutadapt to cut common adapters and primers after following their read trim procedure [54]. This is because after their suggested read trim procedure from the original study, there are still certain samples with an extremely large ratio of the observed occurrence to the expected occurrence of several k-mers at the beginning or end of reads. The results from two different read trim procedures are actually quite similar, because the number of the affected reads is relative small compared with the number of total reads within samples.

Although we redid the analyses with almost the same procedures by the same tool, our result from both versions of MetaPhlAn is quite different from what was reported in the original study (Table 2). With the old version, MetaPhlAn identified one phylum (*Actinobacteria*), one class (*Actinobacteria*), and three species (*Alistipes shahii*, *Clostridium asparagiforme*, *Bacteroides caccae*) with read abundances significantly different between samples (p-value<0.05). The *Bacteroidetes* phylum itself is not significantly different between samples, although two of the three identified species are from this phylum. The only significant phylum identified is *Actinobacteria*, together with one of its classes. With the latest version, MetaPhlAn identified one phylum (Actinobacteria), one class (Actinobacteria), one family (*Rikenellaceae*), one genus (*Alistipes*), and six species (*Alistipes shahii*, *Alistipes finegoldii*, *Alistipes onderdonkii*, *Bacteroides caccae*, *Eubacterium siraeum*, *Eubacterium sp*. *3_1_31*) with read abundances significantly different between samples (p-value<0.05) (Table 2). Similarly, the *Bacteroidetes* phylum itself is not significantly different between samples, although four of the six identified species together with one identified genus and one identified family are from this phylum. The only significant phylum identified is *Actinobacteria*, together with one of its classes. One species identified by the old version is not discovered by the latest version, indicating that multi-reads may affect the downstream analyses and unique reads with current annotation may become multi-reads in the future. We also tried the old version without changing the read trimming procedure in the original research, we still only identified *Actinobacteria* as the only significant phylum between samples (S3 Table). We also studied the correlation of the read abundances of these identified taxa with the colitis severity in patients. All identified taxa have better correlation than *Bacteroidetes* or almost all of their mapped reads are from one type of samples and thus cannot calculate the correlation (Table 2).

**Table 2:** Comparison of results from our analyses and from two MetaPhlAn based analyses.

	taxa reported by the original study	taxa from MetaPhlAn version 2.7.0	taxa from MetaPhlAn version 1.7.7	taxa from our pipeline that are reported by the original study or identified by MetaPhlAn
Phylum	1(*Bacteroidetes*)	1(*Actinobacteria*)	1(*Actinobacteria*)	2(*Bacteroidetes*, *Actinobacte*ria)
Class	0	1(*Actinobacteria*)	1(*Actinobacteria*)	0
Order	0	0	0	0
Family	3(*Bacteroidaceae*, *Rikenellaceae*, *Barnesiellaceae*)	1(*Rikenellaceae*)	0	2(*Rikenellaceae*, *Barnesiellaceae*)
Genus	0	1(Alistipes)	0	1(*Alistipes*)
Species	0	6(*Alistipes shahi*, *Bacteroides caccae*, *Alistipes finegoldii*, *Alistipes onderdonkii*, *Eubacterium siraeum*, *Eubacterium sp*. *3_1_31*)	3(*Alistipes shahii*, *Clostridium asparagiforme*, *Bacteroides cacca*)	2(*Bacteroides caccae*, *Alistipes finegoldii*)

The numbers in the table are the number of significant taxa identified by different pipelines. The names of these taxa are provided in the parentheses.

We compared the results from our analyses in the previous sections with those from MetaPhlAn. Many more taxa are identified by mapping reads to available sequenced genomes than by MetaPhlAn (Tables 1 and 2). The reason may be because MetaPhlAn mapped reads to marker genes, which cannot work well when the number of reads from a taxon is limited. Therefore, the two analyses from MetaPhlAn can only identify certain taxa from the two most abundant phyla. In addition, many taxa identified by MetaPhylAn and by the original study are also discovered in our study, supporting the colitis-relatedness of these taxa. A few taxa discovered by MetaPhlAn and by the original study are not found in our study, suggesting that these taxa may be unreliable.

## Discussion

By mapping metagenomic reads to all available microbial genomes, we identified at least 3 phyla, 2 classes, 9 orders, 22 families, 70 genera and 162 species that are potentially colitis-related (last column of Tables 1 and S1 and S2). This is because the abundance of each of these identified taxa is significantly different between CF and PtC samples, and correlates with the colitis severity in patients better than the abundance of *Bacteroidetes*. Moreover, these taxa are identified by both unique reads and all mapped reads. In addition, 2 phyla, 1 order, 4 families, 18 genera and 71 species are colitis-related based on literature search (S1 and S2 Tables). Compared with the previously identified colitis-related taxa from the same data, we identified much more taxa supported by literature.

We require that the read abundances of potential colitis-related taxa is significantly different between CF and PtC samples, and correlates well with the colitis severity, for both unique reads only and for all mapped reads together. We have lower confidence on the colitis-relatedness of certain taxa such as the *Chlamydiae* phylum, although its abundance of all mapped reads instead of only unique reads is significantly different between samples, and correlates well with the colitis severity. This is because of our assumption that reads are randomly chosen to be sequenced from a genome and there should be more unique regions for a given microbial genome than shared regions with other genomes. Under this assumption, a significant taxon should have unique read abundances significantly different between CF and PtC samples.

We show that multi-reads affect the analysis results. The inferred taxa based on unique reads only are not always consistent with and sometimes quite different from the inferred ones based on all mapped reads. This implies the necessity to develop better methods to accurately assigned multi-reads to the “bona fide” genomes, which cannot be done satisfactorily at present. Moreover, this also calls for cautious consideration when we remove duplicated reads before mapping. Different from read mapping in individual species, where duplicated reads only affect a small portion of repetitive regions, duplicated reads in metagenomics likely affect the analysis of the present species and their abundance, as duplicated reads can be mapped to multiple species as well.

Although we do not have high confidence on the colitis-relatedness of certain taxa because they are insignificant based on unique reads, they can be still biologically significant and related to colitis development. For instance, the *Chlamydiae* phylum is not considered colitis-related in our study. However, its lower taxa at the level of order, family, genus, and species are all significant based on unique reads. The abundance of these significant lower taxa has an average correlation with the severity of colitis around 0.41. One of its lower taxa at the species level, *Chlamydia felis*, has a correlation of 0.415. Although zoonotic infection of humans with *Chlamydia felis* is not reported, *Chlamydia felis* is a bacteria found in cats and is primarily for the inflammation of feline conjunctiva, rhinitis and respiratory problems [55, 56].

We compared our results based on sequenced genomes with those from MetaPhlAn. We identified many more colitis-related taxa based on sequenced genomes (Table 1). The majority of these missed taxa by MetaPhlAn analyses are low abundant. They are missed by MetaPhlAn, likely because there are many fewer reads that can be mapped to marker genes by MetaPhlAn and thus these low-abundant taxa are not different between CF samples and PtC samples. In addition, since the original study was submitted in November 2015, there are 74 (39.15%) and 159 (38.50%) species sequenced in *Bacteroidetes* and *Actinobacteria*, respectively. We found that the read abundances of 5 of the 74 species and that of 4 of the 159 species are significantly different between CF samples and PtC samples (S1 and S2 Tables), which cannot be identified by MetaPhlAn, as the latest version of MetaPhlAn does not include these species. With more sequenced genomes in the future, with our pipeline or with MetaPhlAn, we may identify even more colitis-related species, as there are on average only about 38.65% reads that can be mapped to the sequenced genomes currently (S4 Table). It is worth pointing out that, unexpectedly, different from what the original study reported, the application of two versions of MetaPhlAn shows that *Actinobacteria* instead of *Bacteroidetes* has significantly different abundance between samples (Table 2), suggesting that 16S rRNA read analyses resulted in a different set of taxa from the analyses based on MetaPhlAn. Such an unexpected difference also implies the limitation of 16S rRNA profiling based approaches.

## Conclusion

Our study shed new light on metagenomic studies. It shows the necessity to consider every region in sequenced genomes instead of considering marker genes only. It also suggests caution when working with duplicated reads and multi-reads during the analyses. Moreover, it is mandatory to take into account how newly sequenced genomes affect the results if methods based on sequenced genomes are used. We hope that in the near future, new and better tools to consider multi-read mapping and novel methods without relying on sequenced genomes can be developed so that the issues here can all be addressed or at least minimized.

## Methods

### Data and their processing

Pair-end raw read datasets from ten PtC samples and twelve CF samples were downloaded under the BioProject ID: PRJNA302832. There were 78 files in this dataset, in which only 44 files correspond to shotgun metagenomic reads of the 22 patients. We thus only analyzed shotgun metagenomic reads from these 44 files. The program fastq-dump was used to convert raw read datasets into fastq format. Cutadapt was used to cut common adapters and primers with the command: cutadapt—minimum-length 36 -q 3, 3 -a file: common_adapter. After removing adapters and primers, there were still based pairs at the start and the end of reads with low quality or excessively k-mer content based on fastQC. These base pairs were cut by cutadapt with the command: cutadapt—minimum-length 36 -q 3, 3—cut 10—cut -10 -U 10 -U -10. Finally, seqtk was used to convert fastq to fasta (Fig 1).

### Database preparation and Centrifuge

We mapped the processed reads to sequenced microbial genomes with the Centrifuge tool [20]. Firstly, all possible complete genomes of archaea, bacteria and viruses were downloaded from NCBI (ftp://ftp.ncbi.nlm.nih.gov/genomes/), which include 245, 7410 and 7281 complete genome sequences for archaea, bacteria and viruses, respectively. With the corresponding assembly summary file, we found the taxonomy ID of each complete genome sequence. With the detail taxonomy ID file at ftp://ftp.ncbi.nlm.nih.gov/pub/taxonomy/, we calculated the full lineage information for each complete genome sequence. These sequenced genomes were from 38 phyla, 71 classes, 162 orders, 451 families, 1638 genera, and 9980 species. Secondly, dustmasker was used to mark the low quality reads with command: dustmasker -infmt fasta -in inName -level 20 -outfmt fasta | sed '/^>/! s/[^AGCT]/N/g' > resName. Thirdly, centrifuge-build was used to build the index for Centrifuge with the command: centrifuge-build -p 8—conversion-table seqid2taxid.map—taxonomy-tree nodes.dmp—name-table names.dmp input-sequences.fna abv. Finally, Centrifuge was used to annotate raw reads with the command: centrifuge -f -p 8 -t -x index_name -1 forward.fasta -2 reverse.fasta -S result—report-file result_report. Centrifuge gives score(s) to each mapped read. Reads with more than one score are multi-reads that can be mapped to several genomes (Fig 1).

For a sequenced genome, we counted the mapped reads in each sample and normalized this number by dividing the count by the total number of reads in the corresponding sample. We then compared the twelve normalized numbers from the CF samples with the ten normalized numbers from the PtC samples for this sequenced genome. For a taxon at the level higher than the species level, reads from all species contained in this taxon were counted, normalized, and compared.

### MetaPhlAn

We inferred the present species and the read abundances by MetaPhlAn with its default parameter [17]. Since it was not clear which version of MetaPhlAn was used in the original study [34], we applied the two latest versions (version 1.7.7 and version 2.1.0) of MetaPhlAn to the shotgun metagenomic dataset. The input for MetaPhlAn was the same as Centrifuge, which were raw reads in fasta format (Fig 1). The output from MetaPhlAn was the read abundances for each taxon at each taxonomical level. The sum of the abundance of all taxa under the same level was 100%. Then we fetched the abundances for each taxon in each sample for further analyses.

### Statistical analysis

Mann-Whitney p-values was calculated with R package, in which the two-sided exact p-value with correction was calculated. The correlation of the read abundances with the severity of colitis was calculate by the Spearman-Rank correlation with python2.7 in the scipy.stats package. The severity of colitis scores were obtained from the original study [34].

## Supporting information

S1 Table Taxa identified with unique reads only. (XLSX)

S2 Table Taxa identified with all mapped reads. (XLSX)

S3 Table Taxa identified by two versions of MetaPhlAn. (XLSX)

S4 Table The number of mapped and unmapped reads in the 22 samples. (XLSX)
